# A method for obtaining field wheat freezing injury phenotype based on RGB camera and software control

**DOI:** 10.1186/s13007-021-00821-7

**Published:** 2021-11-26

**Authors:** Xiuqing Fu, Yang Bai, Jing Zhou, Hongwen Zhang, Jieyu Xian

**Affiliations:** 1 College of Engineering, Nanjing Agricultural University, Nanjing, 210031 China; 2 Key Laboratory of Intelligence Agricultural Equipment of Jiangsu Province, Nanjing, 210031 China; 3 Division of Food Systems and Bioengineering, University of Missouri, Columbia, MO 65211 USA; 4 School of Mechanical and Electrical Engineering, Shihezi University, Shihezi, 832003 China

**Keywords:** RGB imaging, Wheat freezing injury, Freezing injury phenotype, Vegetation coverage, Frost damage grade

## Abstract

**Background:**

Low temperature freezing stress has adverse effects on wheat seedling growth and final yield. The traditional method to evaluate the wheat injury caused by the freezing stress is by visual observations, which is time-consuming and laborious. Therefore, a more efficient and accurate method for freezing damage identification is urgently needed.

**Results:**

A high-throughput phenotyping system was developed in this paper, namely, RGB freezing injury system, to effectively and efficiently quantify the wheat freezing injury in the field environments. The system is able to automatically collect, processing, and analyze the wheat images collected using a mobile phenotype cabin in the field conditions. A data management system was also developed to store and manage the original images and the calculated phenotypic data in the system. In this experiment, a total of 128 wheat varieties were planted, three nitrogen concentrations were applied and two biological and technical replicates were performed. And wheat canopy images were collected at the seedling pulling stage and three image features were extracted for each wheat samples, including ExG, ExR and ExV. We compared different test parameters and found that the coverage had a greater impact on freezing injury. Therefore, we preliminarily divided four grades of freezing injury according to the test results to evaluate the freezing injury of different varieties of wheat at the seedling stage.

**Conclusions:**

The automatic phenotypic analysis method of freezing injury provides an alternative solution for high-throughput freezing damage analysis of field crops and it can be used to quantify freezing stress and has guiding significance for accelerating the selection of wheat excellent frost resistance genotypes.

**Supplementary Information:**

The online version contains supplementary material available at 10.1186/s13007-021-00821-7.

## Introduction

Wheat yield level has a direct impact on national food security, because wheat is a main food crop [1, 2]. Low temperature freezing injury is one of the main natural problems affecting wheat growth [3], and it occurs in the seedling stage, which results in losses in the agricultural production [4, 5]. Slight freezing injury leads to different degrees of yield reduction, whereas severe freezing injury will lead to low yield and serious losses in agricultural production [6]. Therefore, the detection of freezing injury is necessary for agricultural breeding and guidance. The traditional estimation of wheat freezing damage area and damage degree usually depends on the naked eye observation and judgment of agricultural experts, but the estimation result has a large error. Gao et al. [7] and Ye et al. [8] determined the effects of low temperature stress on crops by judging the changes of chemical elements in seedlings. However, these methods are time-consuming and laborious; they hinder promotion and application. Therefore, for food security, a rapid and non-destructive diagnostic method of wheat low temperature stress is needed to accelerate the breeding of excellent frost resistance wheat genotypes [9, 10]. Optical method has advantages in rapid nondestructive testing. Therefore, many researchers have tried various optical sensors to evaluate crop growth [11–13]. In terms of freezing damage detection, Li et al. [14] conducted a preliminary study on chlorophyll fluorescence characteristics of cotton seed seedlings under low temperature stress. Wu et al. [9] studied the degree of freezing damage of wheat seedlings in multiple periods by using imaging spectrum and image integration technology, which can accurately reflect the parts of wheat seedlings with freezing damage. For the large-scale measurement of freezing injury, remote sensing can potentially measure freezing injury [15]. Remote sensing monitoring was used to assess the freezing injury of spring wheat, summer maize [16], and winter wheat [17] in Ningxia. To systematically evaluate the severity of winter wheat freezing injury, Wang et al. [18] proposed a grey system model (GSM) to monitor the extent and distribution of winter wheat freezing injury. Zhang et al. conducted remote sensing monitoring research on freezing damage of spring wheat and summer maize in Ningxia [4]. Zhang et al. explored remote sensing technology and methods for monitoring winter wheat freezing injury in Henan province by using NOAA data and meteorological data [5]. These early studies provided the basis for remote sensing monitoring of freezing damage and estimation of food loss, but the remote sensing data used had the problem of low spatial resolution, and the advantages of large-scale detection were not brought into full play. With the rapid development of materials science and the urgent need of natural disaster warning and prediction, high-resolution satellite data are constantly appearing, which provides solid data support for monitoring and evaluation of wheat freezing damage. But these satellite images are expensive and take a long time to operate, limiting timely warning and prediction. Due to the delayed and hidden characteristics of the symptoms of wheat freezing injury, a large number of near field studies are urgently needed. Truss-type phenotypic equipment is widely used for the complex field environment; it has the advantages of high acquisition accuracy, good stability, high imaging quality, and all-day plant monitoring [19, 20]. Therefore, the use of near-ground truss type phenotype detection equipment can be a good solution to the above problems.

Vegetation coverage is an important parameter concerned by agronomists and ecologists, which can reflect the ability of vegetation to capture light and indicate the biological yield of vegetation. Using optical imaging technology to study vegetation coverage has a good performance in the application of precision agriculture [7]. For example, Li et al. used RGB images to extract the coverage of wheat at different growth stages to provide methods and theoretical support for monitoring wheat growth [8]. Lu et al. used machine vision to extract the coverage of winter wheat, analyze the influence of different coverage on wheat canopy spectrum and quantitatively estimate the yield [9]. Wang et al. also extracted the coverage of cotton with RGB camera and estimated the chlorophyll and nitrogen contents of cotton leaves with spectrometer [11]. The above research results indicate that the image technology based on accurate coverage extraction has great advantages in studying the fine changes of crop nutrients, growth trend analysis, stress characteristics extraction and so on. However, there are few previous studies on the characteristics of wheat coverage after freezing stress, and researchers prefer to use meteorological data to explain the impact of freezing stress, but pay little attention to the change of wheat coverage after freezing stress. Therefore, in this paper, based on the near-ground truss phenotype platform, the method of mechanical vision was used to study wheat freezing stress.

The field mobile phenotype cabin is a large-scale phenotype platform that acts on the field environment to realize the functions of real-time climate simulation, full coverage of crop varieties, and remote real-time tracking simulation. The device is based on the RGB freezing injury system, which is composed of a mobile warehouse in the field and an RGB camera. It is used to realize the automatic and efficient acquisition and identification of wheat canopy freezing injury images. In addition, the software of data acquisition control and phenotype extraction was specifically developed to facilitate shooting and operation. In this paper, the RGB images of different wheat genotypes obtained from the phenotypic warehouse in the field were used to analyze and calculate the wheat seedling stage vegetation coverage. Through image processing technology, the freezing injury part of winter wheat was analyzed and identified, and the freezing injury level was divided to provide reference information for wheat breeding and agricultural activities.

## Materials and methods

### Experimental setup

**Fig. 1:**
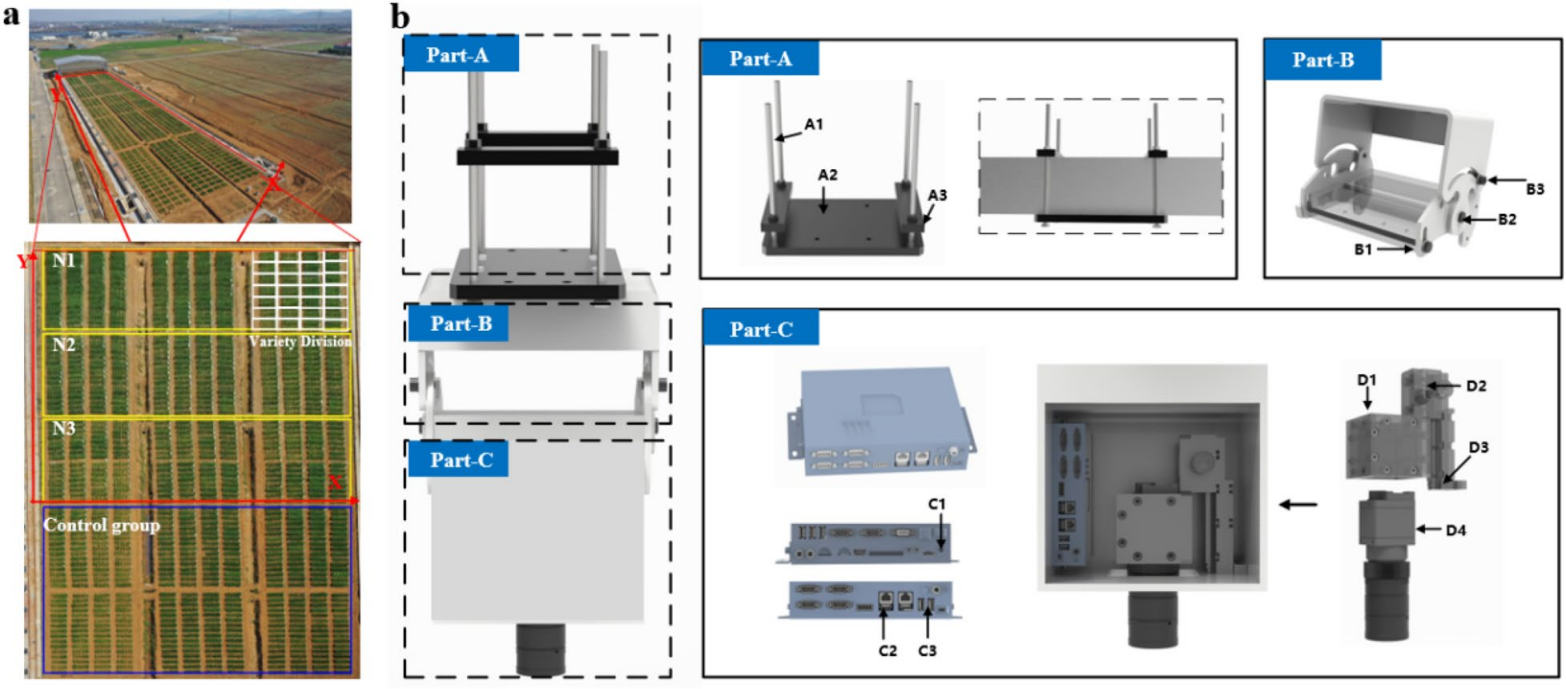
**a** Mobile phenotype cabin in field and wheat regionalization **b** RGB freezing injury system hardware overview

The winter wheat experiment was conducted in Nanjing Agricultural University Baima Teaching and research Base of Jiangsu Province, China (31°36′ 36′′ N, 119° 10′ 48′′ E). The region has a mid-latitude subtropical monsoon climate with simultaneous hot and rainy seasons. The annual precipitation of Nanjing is more than 1000 mm, thereby making it a humid area. The annual average temperature of the whole province is between 13.6 ℃ and 16.1 ℃. In order to minimize the effects of nitrogen application and edge effects, 128 wheat varieties were sown under three nitrogen fertilizer gradients [N1, 0 kg ha^−1^; N2, 180 kg ha^−1^; and N3, 240 kg·ha^−1^ (urea, 46% N)] on November 11, 2020. All wheat individuals were planted in a regular plot. Each district grows a single variety of wheat, 1.5 m long, 1.5 m wide, 6 lines of plants planted in a single small area, and the line spacing is maintained at 25 cm, resulting in a total of 768 plots in the test field. The time of freezing damage occurred 2 months after sowing. The sowing quantity was uniform in all plots, and unified seedling setting was carried out after sowing to ensure the same number of seedlings in all plots, so as to exclude influences such as germination rate. The field moving phenotypic bin and plot layout are shown in Fig. 1a.

The wheat plants experienced server freezing stress at the seedling stage. From Jan 6, 2021, the cold air affected Jiangsu from south to north, resulting in the continuous drop of temperature. From the 6th to the 7th, the cooling range of Baima base was more than 10 ℃, and the lowest temperature fell below – 9 ℃ from the 6th to the 8th. This low temperature had a very wide range of influence and resulted in serious freezing in the whole province, leading to the freezing injury of winter wheat. On Jan 9, 2021, the wheat freezing injury were rated by an experienced breeder rated.

### Hardware component of the phenotyping platform

The overall structure of the phenotyping platform is shown in Fig. 1b. The platform consists of a top connection structure (part-a), a 2D rotating mechanical structure (part-b), and an imaging box (part-c). The top connection adopts a long bolt (A1) to connect the upper connecting plate (A3) and the bottom connecting plate (A2), which are connected to the field mobile warehouse. In the 2D rotating mechanical structure, the head of the imaging box is connected by fixed axis (B2), rotated by rotating axis (B1), and locked by B3 to ensure an 120° imaging angle. An industrial computer (C), in the imaging box receives instructions from the network to control the camera to take photos and sends back the download address of each photos. C1, C2, and C3 are connected to the power supply, the camera, and the computer, respectively. A industrial RGB camera (D4, Model: MV-CA050-20GC, brand, manufacturer location) is fixed on the camera fixed bracket (D1) and connected to the fastening pulley (D2) fixed on the D3 slide rail. This rail can allow the adjustment of the vertical height to realize the adjustment of the camera shooting distance. In this study, the camera was positioned to take images at a nadir view at 1.5 m above the ground. Detailed parameters of the camera can be found in Additional file 1: Table S1.

The overall shell of the machine body is made of an aluminum profile, which is lightweight but has high strength and hardness. A power supply (Uninterruptible Power Supply) is used to adjust the voltage sensitivity, detect the abnormal line voltage, and automatically switch the battery operation to protect the connected equipment.

### Software components

**Fig. 2:**
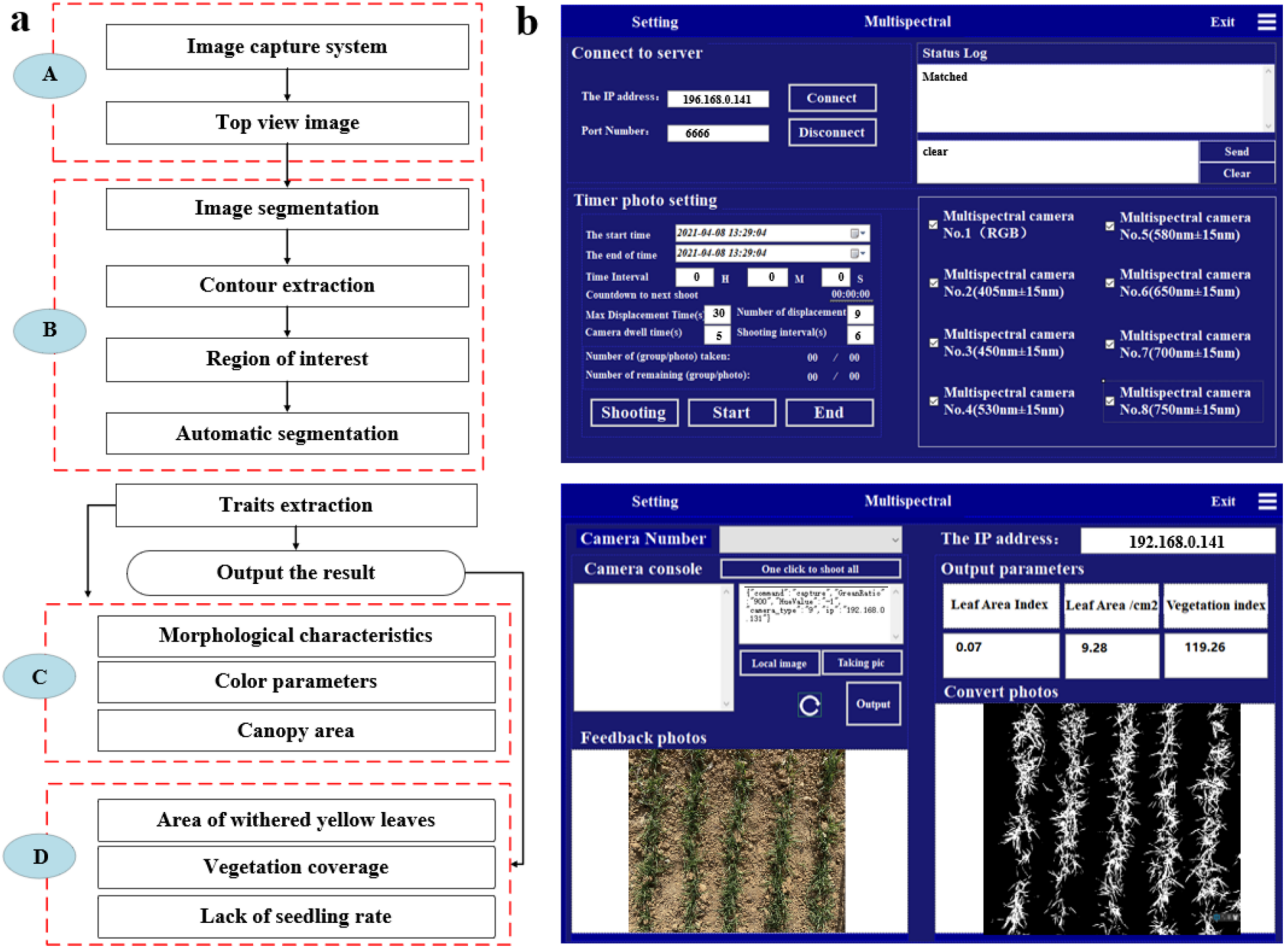
**a** The image processing pipeline **b** Software interface of the phenotyping system

The hardware system needed to be equipped with a development software. The software acquisition and analysis system required Visual Studio 2019 to write the interface and MATLAB to write the analysis algorithm. The capture control of the image was performed. Fine tuning of the view was done through the image return, thereby obtaining the device information and achieving the best size and parameter configuration. Moreover, the RGB image was captured and displayed. The captured image was automatically saved to the specified folder, and the processed binary image was also saved to the specified address for further calculation and research. The software interface is shown in Fig. 2b.

### Image processing

Figure 2a shows the image processing flow, which includes the following four steps. (A) Image acquisition of wheat canopy freezing injury. The top view of crop canopy was obtained by hanging the device at a suitable height. The height of the image acquisition should be consistent. Three RGB images were taken for each nitrogen content gradient and wheat species plot, and a total of 2304 pictures were taken. The time of image shooting was selected under actual natural light in the field from 12 to 3 pm, and the light intensity of the whole shooting process was 2750–2850 LX. (B) The obtained image was preprocessed first in the image segmentation. Then, the binary image was segmented, the contour was extracted, and the region of interest was obtained. (C) Image feature extraction was performed through binary segmentation of the image. The target region pixel and related color features were obtained. (D) The canopy structure parameters were calculated, and the degree of freezing injury of wheat was obtained.

### Image segmentation

**Fig. 3:**
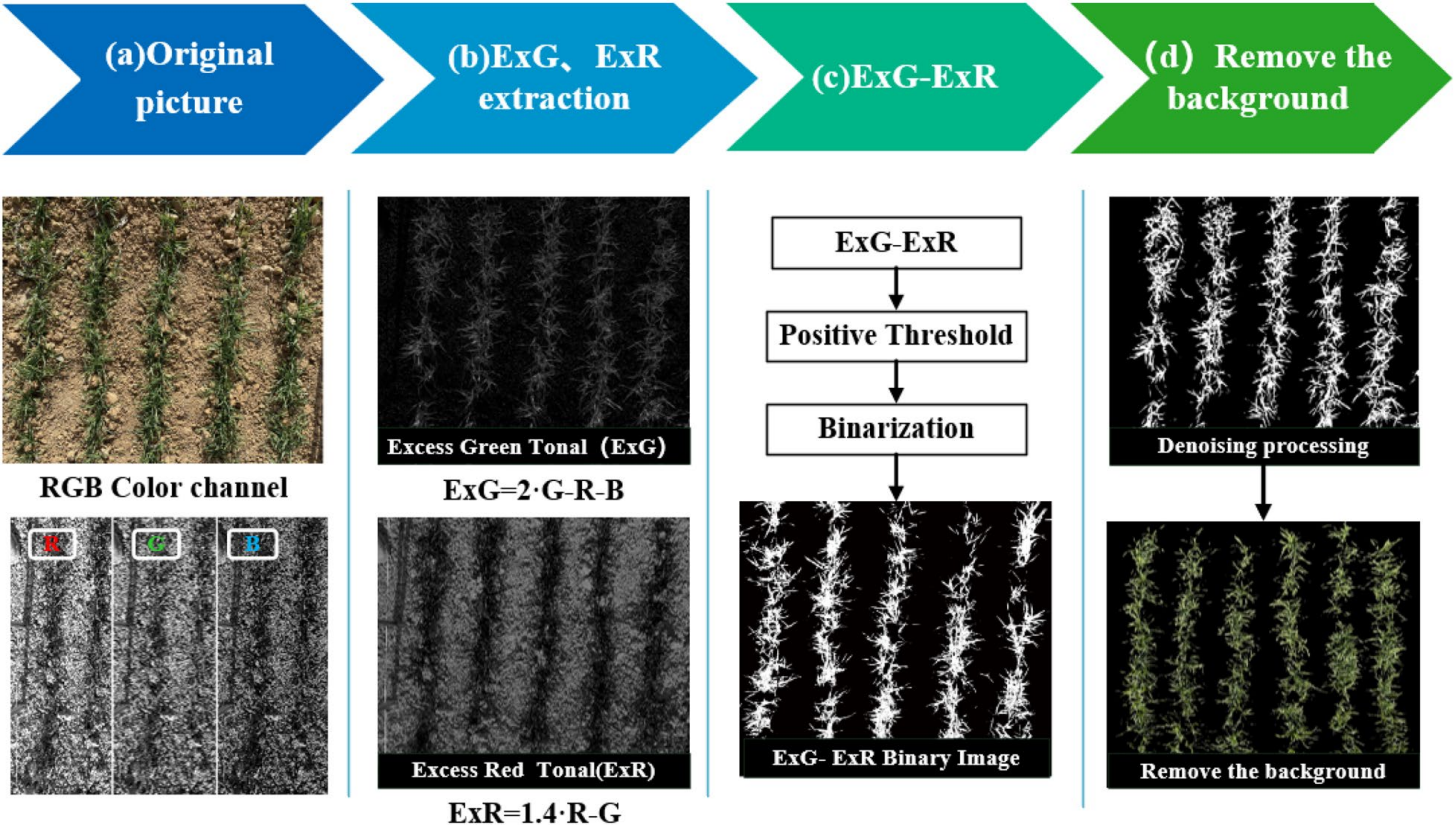
RGB image segmentation process

For the collected images of wheat freezing injury, the values of R, G, and B of green plants and background soil had different characteristics [21]. By separating the original image into three independent primary color planes and then selecting different color feature combinations, each pixel in the image was converted to enhance the contrast between the target crop and the background soil in the image. The extraction method of over green index minus over red index satisfactorily identified the green plants in the image; suppressed the shadow, withered grass, and soil background; and highlighted the green plants in the image. The segmentation process is shown in Fig. 3. First, the Otsu [22] method was used to automatically select the threshold value of gray data. Then, the gray value of the super green minus the super red component of each pixel was calculated and compared with the threshold value. According to the comparison result, the pixel was divided into plants or background. The color feature values to be extracted included excessive green (ExG) [23], excessive red (ExR) [24], and ExV [25].

1 $$ExG = 2g - r - b$$

2 $$ExR = 1.4r - g$$

3 $$ExV = ExG - ExR = 2g - r - b - 1.4r + g$$

In the above formula, r, g, and b are the normalized pixel values of the red (R), green (G), and blue (B) channels, i. e., r = R/(R  +  G  +  B), g  =  G/(R  +  G  +  B), and b  =  B/(R  +  G  +  B), respectively.

The binarized image was obtained by threshold segmentation, and then, the ROI contours containing all the plants were extracted. The cv2.bitwise_and function was called in OpenCV, and the mask was applied on the binary data (binarized picture), retain the white area (plant) image pixels of the mask image, and remove the black area (background) image pixels to complete the image segmentation by removing the background.

### Image feature extraction

Vegetation coverage (VC) is an important indicator to measure the change of vegetation growth status on the land surface. It usually refers to the percentage of the total area of vegetation (leaves, stems, and branches) projected vertically on the ground to the total area of the statistical area. VC reflects the photosynthesis capacity of plants and the growth status of plants [26]. When using this system to collect canopy vertical angle image, we should try to avoid using the widest or farthest shot end of the lens focal length and use a smaller aperture to reduce the geometric distortion of the image. After image processing, the background of wheat image was removed, and the proportion of green pixels of wheat to the total pixels was calculated by using the segmented gray image, i. e., the proportion of wheat vertical projection area to the total area. The result was the wheat coverage of the study area. Considering that the coverage detected was the proportion of the green part, and the wheat freezing injury resulted in shrinkage, the bud tip was yellow and white. Thus, the area of the freezing injury part was not included in the coverage range. The coverage can reflect the wheat growth after freezing injury and can effectively characterize the degree of wheat freezing injury.

### Statistical analysis

Statistical analysis was conducted using the SPSS statistics packages (IBM SPSS statistics 26, Inc., Chicago, IL, USA). A two-way analysis of variance (ANOVA) was to evaluate the effects of nitrogen treatments (N1, N2 and N3 as fixed factors) on the vegetation coverage, leaf area, green index and withered leaf area of wheat varieties (random factors).

ANOVA analyses were also conducted to see if there is significant differences in the image features among the wheat freezing injury rates. To determine the coverage relationship between the cold resistant and non-freeze resistant genotypes, the mean values were compared with the mean values of Least Significant Difference (P  < 0. 05).

## Results and discussion

### Performance analysis of wheat freezing injury

Early winter freezing damage is wheat freezing damage in early winter, usually caused by sudden and strong temperature drop. From mid-late November to mid-December, the lowest temperature plummets about 10 ℃ to below – 10 ℃ for 2–3 days. Wheat seedlings have not been cold resistance exercise, and their cold resistance ability is poor, which is easy to form early winter frost injury. In this paper, planting freezing injury belongs to typical early winter freezing injury, and the types of wheat suffering from freezing injury are weak seedling and flourishing seedling. Strong seedling generally does not cause freezing injury, but mostly causes freezing on leaf tip, which has little influence on wheat growth. The young and weak seedlings, which have not accumulated a lot of soluble solids and are still in the vigorous growth stage, have poor resistance to low temperature and are prone to early winter freezing injury, resulting in leaf drying and seedling death. Early sowing and flourishing seedlings, freezing damage mainly caused by young ear freezing to death and leaf drying, especially in wheat fields with low soil fertility, poor land preparation quality and soil moisture shortage, such as sudden strong cooling weather, easy to cause freezing damage in early winter [27]. Specifically, the leaves turn yellow at low temperatures. The leaves were deformed and dehydrated. The wheat shoots, blown by the wind and burnt by the sun, turn dry white or yellowish white. Under sun exposure, the leaves are folded, bent, and then gradually dried, which is consistent with freezing injured [2] plants. In addition, the effect of different seedling emergence rate can be excluded after seedling reduction treatment in this test field. Therefore, freezing damage is the main research variable in this paper.

### Differentiation of freezing injury degree by vegetation coverage

Studies have shown that coverage change can well reflect vegetation growth status and is an important parameter for nondestructive monitoring of vegetation life information [14]. Crop coverage changes significantly with the development of growth period, that is, it presents a low–high–low trend. After the freezing stress of wheat, the leaves of wheat seedlings were dry and yellow, and the plants appeared dry and withered, and the ground coverage decreased. Normal wheat showed curly leaves, but had life activity. There was no significant change in the size of wheat plant, but there was a significant difference in the ground coverage of wheat. The wheat soil exposed to freezing stress, and the surface became dry and hard. This provides reliable data support for the study of wheat freezing stress by using image technology.

**Fig. 4:**
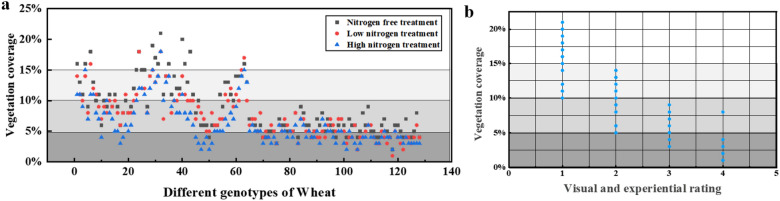
**a** Comparison of vegetation coverage of different wheat genotypes under the three nitrogen treatments. **b** Box diagram of vegetation coverage and visual experience frost damage classification

The Fig. 4a shows the coverage levels of 128 genotypes of winter wheat under the conditions of no nitrogen application, low nitrogen application, and high nitrogen application. In general, in the comparison of wheat subjected to treatment with three nitrogen levels after freezing injury, wheat growth in the high-nitrogen fertilizer plot was not good; in 87.4% of the varieties, high nitrogen treatment led to the lowest coverage. The wheat that did not receive nitrogen fertilizer (24.8 kg ha^−1^ nitrogen concentration) grew better than the others. Among 62.2% of the varieties, the wheat with no nitrogen fertilizer showed the best coverage. Figure 4a shows that the coverage of the experimental plot was distributed in the range of 0–20%. The highest vegetation coverage rate was 21%, and the lowest was 2%. The coverage difference between various species was obvious, which reflected the degree of resistance of different species to freezing injury.

**Fig. 5:**
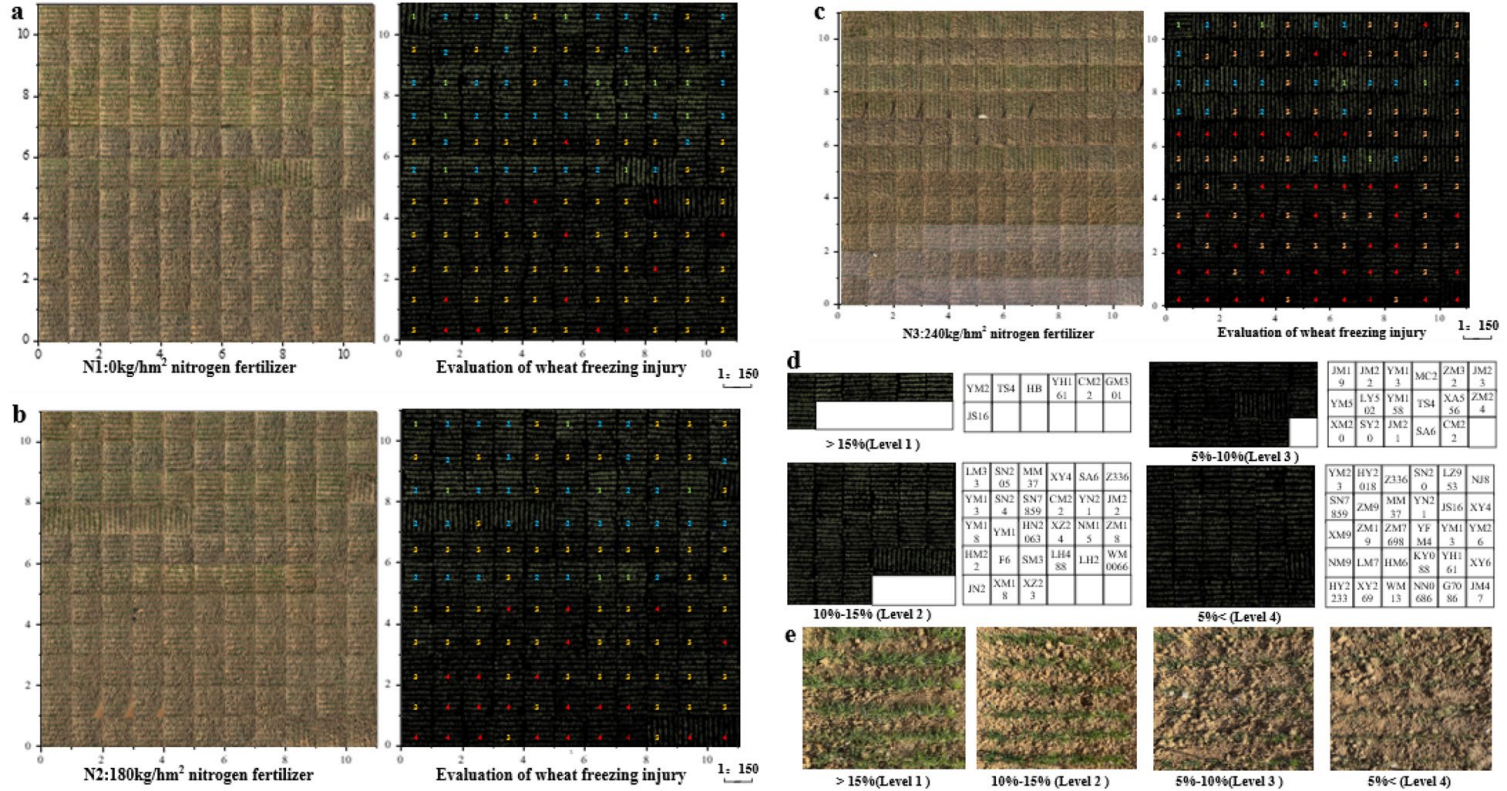
Frost damage rating of wheat in the following: **a** physical map of n1, 0 kg/hm^2^ nitrogen fertilizer treatment and wheat grading of different genotypes; **b** physical map of n2, 180 kg/hm^2^ nitrogen fertilizer treatment and wheat grading of different genotypes; and **c** physical map of n3, 240 kg/hm^2^ nitrogen fertilizer treatment and wheat grading of different genotypes. **d** Classification of freezing injury in different wheat varieties **e** Original pictures of different levels of coverage

According to the evaluation standard of wheat freezing injury (Additional file 1: Table S2), 128 kinds of field wheat were classified. The differences between the image factors and the degree of freezing injury were analyzed by variance analysis. Significant differences exist in the average vegetation coverage of 128 wheat varieties at the budding stage, and different nitrogen treatments had significant effects on the frost resistance of wheat, especially the significant correlation between the coverage and the evaluation of frost damage (Additional file 1: Table S3). Figure 4b presents a box chart of vegetation coverage and wheat frost damage classification, which showed that the coverage demonstrated a good distinction between plants with frost damage and those without frost damage. Therefore, the coverage was selected as the main division segment in this paper. Based on the coverage, different genotypes of wheat were classified and graded. The four intervals were as follows:  < 5% (severe freeze injury area); 5–10% (severe freeze injury area); 10–15% (slight freeze injury area); and  > 15% (no obvious freeze injury area). The physical images of different levels and the segmented images are shown in Fig. 5. Based on this method, the frost damage grades of 128 genotypes and three nitrogen treatments were determined. The four intervals corresponded to 1, 2, 3, and 4 grades. Grade 1 represented the lightest injury, and grade 4 represented the most serious. The growth of different genotypes of wheat in extreme cold weather can be clearly seen through the field plot rating screening presented in Fig. 5a, b, c. The excellent genotypes (grade 1 freezing injury) calibrated by this method included HM 33, ZM 16, and LM 26(Classification of freezing damage of 128 varieties as shown in Fig. 5d). Further investigation showed that these wheat plants had strong cold resistance, resisted low temperature, and belonged to varieties with satisfactory cold resistance. Similarly, through the screening of the rating method, it was found that the cold resistance of wheat varieties with higher freezing injury level was generally weak. Therefore, this method can provide a scientific basis for the screening of wheat varieties with freezing injury resistance; it can also provide agricultural production guidance, reduce the dead seedling rate, and improve the yield.

### Precision evaluation of freezing damage grade

**Fig. 6:**
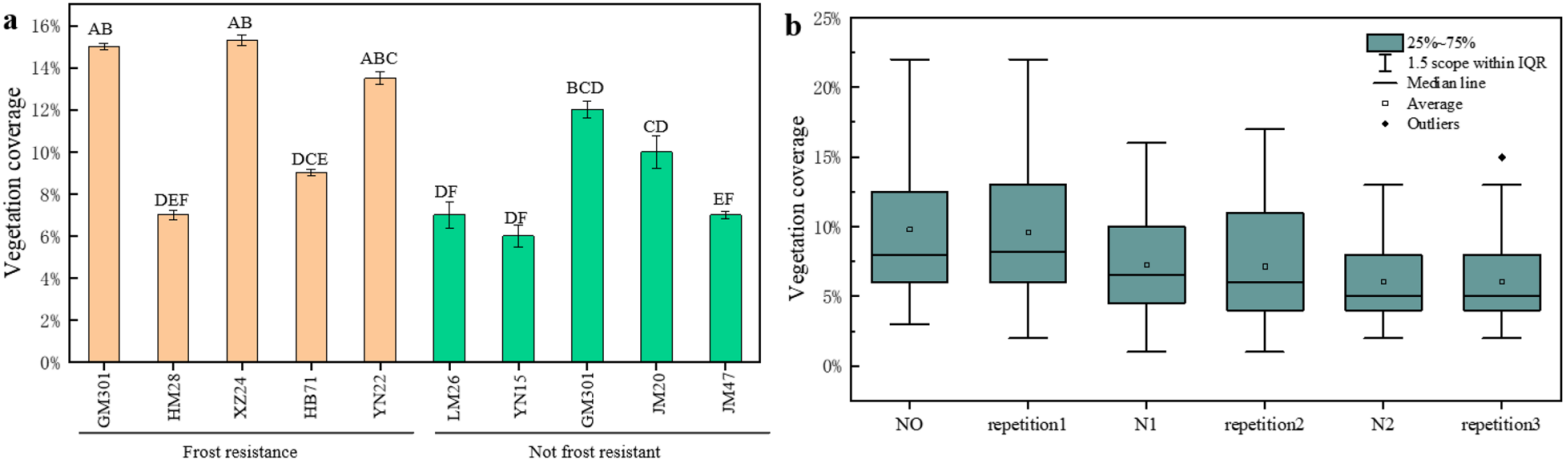
**a** There were significant differences in vegetation coverage between representative frost resistant and non-frost resistant genotypes. Different letters on the bar graph indicate significant differences, as determined by LSD (P  ≤  0.05). **b** Biomass repeats (three nitrogen gradients) and boxplots of technical repeats. Corresponding to 128 wheat varieties under 3 nitrogen treatments of two biomass repeats and two technical repeats respectively

A significant correlation existed between the coverage of most wheat genotypes and frost resistance, but there were some special cases. In this paper, the following representative genotypes of wheat were selected to study the correlation of their coverage, the experiment was repeated three times under the control parameters. Although the vegetation coverage varied among genotypes in the frost resistant group, the vegetation coverage of non-frost resistant genotypes was either significantly lower than or not significantly different from that of frost resistant genotypes (Fig. 6a). Thus, the system could only distinguish the obvious frost damage. Figure 6b shows that the biomass repetition mean of 128 wheat varieties with three nitrogen fertilizer gradients is equal without significant difference under two technical replicates, indicating that the system has high accuracy. And the wheat mulch degree under no nitrogen application was significantly higher than that under low nitrogen and high nitrogen.

### Other freezing damage evaluation methods

**Fig. 7:**
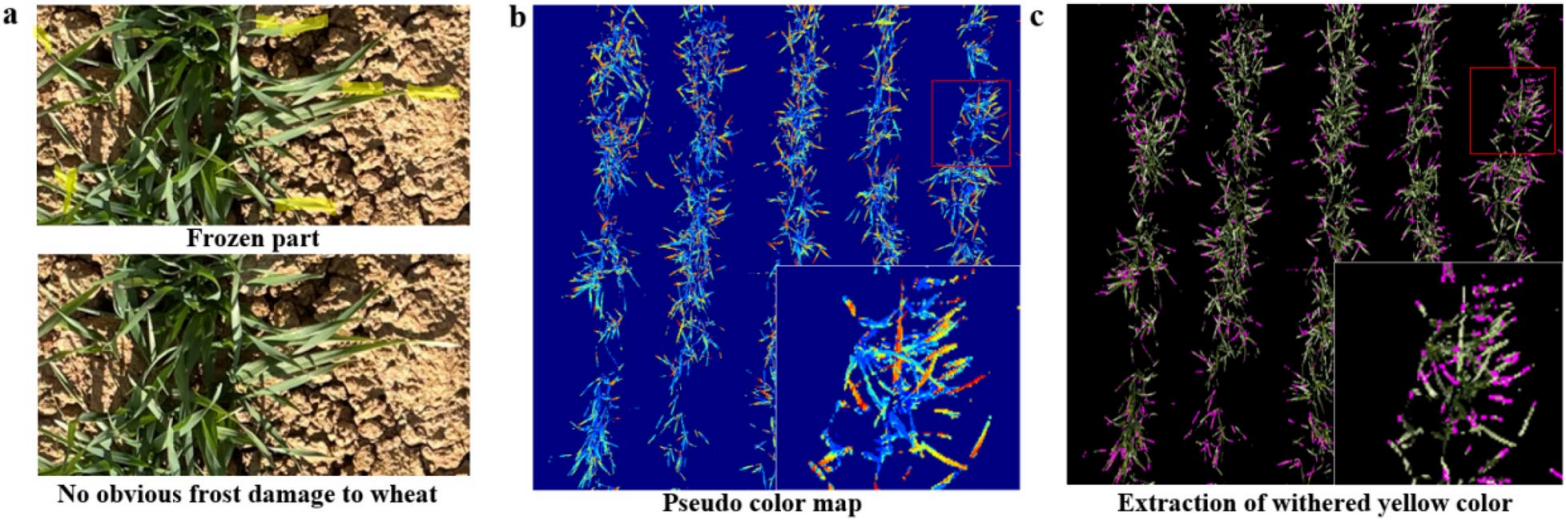
Pseudo color map of wheat in light freezing injury area, as follows: **a** freezing injury area, **b** withered yellow leaf extraction, **c** actual image of withered yellow leaf extraction

Through field observation, freezing damage was found to also exists in the plot with high coverage, as shown in Fig. 7a. The yellow area in the Figure showed freezing damage. The leaf tips withered and became yellow and white. The high coverage plot grew well. Thus, the frost damage area was small, and the injury was concentrated on the leaf tip. Compared with the overlapping situation, the low coverage plot better identified the frost damage area by color. In this experiment, the image was converted to HSI channel to extract the threshold color, and the withered leaves of high coverage area were extracted, as shown in Fig. 7b (the pseudo color image) and Fig. 7c (withered leaves extraction). We found that this extraction method had some deviations, such as sunlight reflection, similar background color, and yellowing of bud color, but it was still able to reflect a certain degree of freezing injury.

### The advantages and disadvantages of this detection system compared with other monitoring methods

With the development of various detection technologies, different phenotypic tests have inconsistent requirements for flux, input cost, platform benefit, monitoring accuracy, etc., which requires various types of sensors to address the diverse needs. The advantages and disadvantages of different types of phenotypic platforms are shown in Additional file 1: Table S4.

Since wheat seedlings are small in the high frequency period of reversed spring cold, there are some problems such as low spatial resolution, low accuracy and large deviation of measurement results by using uav. Moreover, the detection of freezing injury is characterized by delay and concealment, and the low stability of airborne phenotype platform results in poor data acquisition accuracy. This system is an outdoor truss near-ground phenotype detection platform, which can overcome the influence of environmental lighting and other factors, and achieve a highly automated, low-cost, high-precision plant freezing injury phenotype scheme.

## Conclusion

This paper discussed the possibility of using the micro phenotype information acquisition platform with RGB camera and remote control as a mobile phenotype analysis technology. In this experiment, automatic image segmentation and character extraction were used to promote the study of plant phenotypic information. The RGB to HSV color space and color threshold extraction can effectively remove the image background. At the same time, binarization is used to calculate the leaf area coverage rate, seedling shortage rate, and other characteristics.

This system is suitable for near-earth plant early frost damage study, especially in the bud, because wheat freezing injury often occurred in the growth period in the over-wintering stage, green returned stage and jointing stage and booting stage, basically belongs to the early stage of the birth, the coverage is low, the ground of bare soil and usual crop straw, drought and other factors, coverage factors affecting complex; In addition, previous scholars tend to use meteorological data to explain the impact of freezing damage, but in this case, the shooting accuracy of UAV is not accurate enough to distinguish, and the near-ground RGB freezing damage system can promote the research on freezing damage in such cases.

- (1) The basic software and hardware system selection of phenotype acquisition equipment, including the design of the structure and control system, and a software system for shooting calculation, was developed for this equipment. This equipment can be used for camera control shooting and image preliminary analysis.
- (2) To further explain the operation principle of the system, the operation process and data acquisition principle are interpreted one by one, including image processing process, segmentation method, and coverage acquisition method.
- (3) In addition, through data collection, the coverage of 128 different genotypes of wheat were extracted, and the grade of freezing damage was classified.
- (4) Finally, we compared the advantages and disadvantages of different freezing injury equipment and analyzed the prospect of combining RGB freezing injury sysytem with other freezing injury detection methods in the future.

In general, RGB freezing injury system provides a high throughput phenotypic research platform and a solution for wheat freezing injury analysis. Compared with the same type of system, this program has the advantages of high pertinence, high stability, higher precision of data acquisition, and can collect phenotypic data with high automation. At present, most of the detection methods of freezing damage are satellite images, unmanned aerial vehicles and hand-held ones, while the detection methods of near-ground moving freezing damage are few. Therefore, as a means of low Earth orbit detection, this system has a certain research significance in the exploration of freezing damage. Through the rapid determination of the grade of freezing injury in RGB freezing injury system, cold resistance can be identified, cold-resistant varieties can be bred, or targeted and timely remedial measures can be taken according to the situation of freezing injury to reduce losses, so it has a certain contribution to agricultural production.

## Supplementary Information

**Additional file 1: Table S1.** Key performance parameters of RGB camera. **Table S2.** Design parameters of wheat field experiment. **Table S3.** Evaluation of freezing injury degree and analysis of variance of main effect mean of different factors. **Table S4.** The advantages and disadvantages of this system are compared with other platforms

## Data Availability

Not applicable.
