# Supplementary material for: A method for obtaining field wheat freezing injury phenotype based on RGB camera and software control
Source: Plant Methods. 2021 Nov 26;17:120. doi: 10.1186/s13007-021-00821-7 (PMC8620711; doi:10.1186/s13007-021-00821-7)
Supplement: Supplementary file 1 — Additional file 1: Table S1. Key performance parameters of RGB camera. Table S2. Design parameters of wheat field experiment. Table S3. Evaluation of freezing injury degree and analysis of variance of main effect mean of different factors. Table S4. The advantages and disadvantages of this system are compared with other platforms [file 13007_2021_821_MOESM1_ESM.docx]

**Supplementary**

**Table S1.** **Key performance parameters of RGB camera**

| **Performance parameters** | **Parameters** | **Parameter setting** | **Parameters** |
| --- | --- | --- | --- |
| Spectrum range | 390nm~780nm | ISO | 100 |
| Resolving power | 3024×4032 | color temperature | 4900K |
| Pixel spacing | 4.8µm | time of exposure | 1/6~1/5s |
| Detector frame rate | 22 fps | aperture | 2.4 |

**Table S2. Design parameters of wheat field experiment**

| **Degree of freezing injury** | **Classification standard /characteristics** |
| --- | --- |
| Level 1 | Less than 1/4 of the leaves were dry |
| Level 2 | Less than 10% of the seedlings died, and 1/4 -1/2 of the leaves were dry |
| Level 3 | The death rate was 10% - 30%, and 1/2-3/4 of the leaves were dry |
| Level 4 | The death rate was 30% - 50% and 3/4 of the leaves were dry |
| Level 5 | More than 50% of the seedlings died, and all the leaves dried up |

**Table S3. Evaluation of freezing injury degree and analysis of variance of main effect mean of different factors**

| **Test** | **Analysis of variance effect** | **Significance** |
| --- | --- | --- |
| Vegetation coverage | Treatment of different nitrogen levels | <0.001*** |
|  | Genotype(random) | <0.001*** |
| Green index | Treatment of different nitrogen levels | <0.001*** |
|  | Genotype(random) | 0.996 |
| leaf area | Treatment of different nitrogen levels | 0.001*** |
|  | Genotype(random) | <0.001*** |
| Proportion of withered leaves | Treatment of different nitrogen levels | <0.001*** |
|  | Genotype(random) | <0.001*** |
| Vegetation coverage | Visual and experiential rating | <0.001*** |
|  | Treatment of different nitrogen levels | <0.001*** |
|  | Genotype(random) | <0.001*** |
| Green index | Visual and experiential rating | <0.001*** |
|  | Treatment of different nitrogen levels | <0.001*** |
|  | Genotype(random) | 0.936 |
| leaf area | Visual and experiential rating | <0.001*** |
|  | Treatment of different nitrogen levels | <0.001*** |
|  | Genotype(random) | 0.581 |
| Proportion of withered leaves | Visual and experiential rating | 0.475 |
|  | Treatment of different nitrogen levels | <0.001*** |
|  | Genotype(random) | <0.001*** |

*,**,***indicate significance at p = .05, .01, and .001 levels, respectively.

**Table S4.** The advantages and disadvantages of this system are compared with other platforms

| **Planform types** | **Advantages** | **Limitations** |
| --- | --- | --- |
| Indoor fixed platform | High precision, good repeatability | High cost, low single plant detection efficiency, small flux |
| Outdoor doorframe platform | High automation, real-time monitoring, high precision | The acquisition Angle is limited, the cost is high and the monitoring area is fixed |
| UAV Phenotype platform | High throughput and low cost | Large influence of environmental factors, airspace control, small load, low precision |
| Airborne phenotype platform | Large load, high efficiency and flexibility | High cost, low load, low flux, low acquisition accuracy |
